# Functional diversity of soil macrofauna may contribute to microbial community stabilization under drought stress

**DOI:** 10.3389/fmicb.2025.1597272

**Published:** 2025-06-13

**Authors:** Diana Morales-Fonseca, Sandra Barantal, François Buscot, Stephan Hättenschwiler, Alexandru Milcu, Johanne Nahmani, Emmanuel S. Gritti, Kezia Goldmann, Luis Daniel Prada-Salcedo

**Affiliations:** ^1^Department of Soil Ecology, UFZ-Helmholtz Centre for Environmental Research, Halle (Saale), Germany; ^2^Faculty of Engineering, Department Chemical Engineering, Universidad de América, Bogotá, Colombia; ^3^Ecotron Européen de Montpellier, Univ Montpellier, CNRS, Campus Baillarguet, Montferrier-sur-Lez, France; ^4^CEFE, Univ Montpellier, CNRS, EPHE, IRD, Montpellier, France

**Keywords:** mesocosms, Ecotron, abiotic stress, climate change, 16S Illumina sequencing, bacterial resilience, drought-tolerant genes

## Abstract

The impacts of climate change, particularly the increasing frequency and intensity of severe droughts, pose significant threats to terrestrial ecosystems. To ensure the maintenance of critical ecosystem functions under these conditions, it is essential to better understand the interactions between different soil communities. However, the extent to which drought-induced changes in microbial communities are influenced by soil biodiversity, especially the functional diversity of soil macrofauna, remains poorly understood. In this study, we investigated how microbial communities respond to contrasting levels of macrofauna functional diversity and to more severe and prolonged drought in a Mediterranean forest ecosystem, all under fully controlled conditions. We conducted a two-year mesocosm experiment using 16 large mesocosms at the Montpellier European Ecotron, employing 16S amplicon sequencing and inferred functional gene annotations to assess microbial responses. Our results revealed that the relative abundance of Gram-positive bacterial communities increased compared to Gram-negative ones in response to drought. Furthermore, higher levels of macrofauna functional diversity appeared to help stabilize microbial diversity and community composition during periods of severe and prolonged drought. The resilience of microbial communities to drought was further reflected by the enrichment of drought-tolerant genes in specific bacterial taxa. Overall, these findings highlight the importance of preserving soil biodiversity as a means to mitigate the effects of future droughts on soil functions and to enhance the resilience of terrestrial ecosystems in the face of ongoing climate change.

## Introduction

Soil biological diversity plays a critical role in regulating and supporting ecosystem services (Wagg et al., 2014). High diversity of soil macrofauna, including earthworms, millipedes, and woodlice, is often associated with well-structured soils that tend to have high biological activity and relatively stable biogeochemical cycling over time and space (Bhaduri et al., 2022; Ruiz et al., 2008). Such soils are characterized by a rather equilibrated water, air, and organic matter content, adequate nutrient availability, and high biological diversity—all of which supporting high levels of biogeochemical cycling (Lehmann et al., 2020; Salomon and Cavagnaro, 2022). Soil moisture, for instance, affects behavior and abundance of soil fauna, which subsequently impacts nutrient availability, mineralization, and microbial activity (Tsiafouli et al., 2005). Soil fauna are major contributors to decomposition, playing a central role in soil food webs by accelerating litter decomposition through fragmentation, production of fecal pellets (Joly et al., 2020), and grazing on soil microorganisms (Crowther et al., 2012; Hugerth and Andersson, 2017; Ineson and Anderson, 1985). In turn, microorganisms are the major drivers of biogeochemical cycling by mineralizing organic matter, thereby fueling the carbon (C) cycle and providing plant-available inorganic nutrients (Scholle et al., 1992). Accordingly, both macrofaunal and microbial communities influence resource availability in soils (Watzinger et al., 2023).

Macrofauna encompasses the largest soil invertebrates (≥2 mm), including annelids, arthropods, mollusks, and insect larvae, which play essential roles in soil structure and ecosystem functioning (Coleman et al., 2023). The functional diversity of soil macrofauna can also influence aboveground communities (Setälä et al., 1998), with potential cascading effects on microbial abundance and function (Coulis et al., 2015; Heemsbergen et al., 2004; Luan et al., 2024; Lubbers et al., 2020). While these organisms ingest microorganisms, their role in habitat modification and dispersal has the most significant short-term effects on microbial communities (Thakur and Geisen, 2019). In contrast, habitat formation by macro- and mesofauna is responsible for more pronounced long-term changes in microbial community composition (Scheu et al., 2005). Earthworms have an important effect on soil microbial communities depending on the respective species, which can, for instance, increase the abundance of specific bacterial groups (Proteobacteria/Actinobacteria) after passage through the earthworm gut (Yang et al., 2024). Moreover, their movements facilitate the dispersal and distribution of soil microbes and selective grazing on microbial biomass (Medina-Sauza et al., 2019). In the case of arthropods, their activities can modify soil structure, thereby influencing microbial diversity (Wu et al., 2014).

Changes in biological diversity can significantly affect biogeochemical transformations, nutrient availability, and the soil’s capacity to cope with abiotic and biotic constraints (Coulis et al., 2015; Heemsbergen et al., 2004; Luan et al., 2024; Lubbers et al., 2020). Variations in the relative abundance and diversity of soil macrofauna can impact microbial community structure due to their involvement in nutrient mineralization processes, which in turn affects nutrient acquisition and plant growth (Cole et al., 2006; Coleman et al., 2023; Medina-Sauza et al., 2019). Despite the multiple interactions between macrofauna and microbes, there is limited knowledge about how high taxonomic or functional diversity in soil fauna relates to microbial communities and correlates with high abundance or biological activity. Hence, there is a growing interest in understanding the factors that influence and regulate the relationship between soil fauna and soil microorganisms, and the consequences for ecosystem functioning.

Climate change is causing more frequent and severe drought events, which may have strong constraining effects on plants, soil macrofauna, microorganisms, and their interactions (Martin et al., 2024; Peng et al., 2022; Valencia et al., 2018). Drought alters the activity, structure, and functional composition of soil microorganisms, which might impact ecosystem functioning and decrease plant productivity (Li et al., 2023; Seaton et al., 2022; Sheik et al., 2011). Most notable are changes in the relative abundance of microorganisms, typically related to microbial group-specific strategies to respond to drought (Metze et al., 2023). For instance, an increase in the ratio in Gram-positive to Gram-negative bacteria results from a higher degree of tolerance and resilience to the abiotic stress associated with drought conditions of Gram-positive traits (Gillespie et al., 2023; Naylor and Coleman-Derr, 2018). Microbial responses to drought include changes in enzyme activities, nutrient fluxes, and alterations in carbon (C) and nitrogen (N) cycling processes (Bogati and Walczak, 2022). The expression of particular enzyme groups, such as kinases and antioxidants, is also part of drought response strategies, which support adaptive mechanisms during severe drought periods (Abdelaal et al., 2021; Rajpurohit et al., 2022; Sebai and Abdallah, 2022). For example, when cells face a decreased water potential, osmotic stress, desiccation, and high temperature, the respiratory rates increase to compensate the demand of metabolic energy (Rahman et al., 2021). The regulation of gene expression during these responses is related to kinases, which are also involved in DNA protection (Rajpurohit et al., 2022). Likewise, high respiratory rates lead to the production of reactive oxygen species, which are mitigated through the set of enzymes such as catalases that allow the elimination of these compounds (Solana García Andrea, 2021). Nevertheless, the extent to which drought-induced changes in microbial communities are influenced by soil biodiversity, particularly the functional diversity of macrofauna decomposers, remains poorly understood. Addressing this issue is crucial for developing management strategies aimed at enhancing ecosystem resistance and resilience to drought.

In this context, the aim of this study was to evaluate how increasingly severe drought events affect the diversity and composition of soil prokaryotic communities, particularly bacteria, and whether this was modulated by soil macrofauna functional diversity. To this end, using a Mediterranean forest understory model system, we conducted a large mesocosm experiment in a controlled-environment ecotron facility with a fully factorial crossed design that combined a Drought treatment (typical Mediterranean seasonal drought vs. more severe and prolonged drought) with a macrofauna functional diversity treatment (low vs. high functional diversity). We characterized the bacterial communities in the top soil, and predicted the genes related to abiotic stress responses within these communities. We hypothesized that: (i) more severe and prolonged drought conditions will have a stronger negative impact on Gram-negative than Gram-positive bacterial communities; (ii) higher functional diversity of soil macrofauna will increase microbial diversity; (iii) higher functional diversity will lead to a distinct microbial community composition compared to low functional diversity; and (iv) changes in microbial communities will be reflected in their functional capacities to face drought conditions, specifically by increasing the abundance of drought-related enzymes under higher functional diversity of soil macrofauna.

## Materials and methods

### Mesocosm setup

The experiment was conducted at the Montpellier European Ecotron (CNRS, Montpellier, France) in the Mesocosms experimental platform,^1^ whose capabilities were briefly described by Roy et al. (2021). The experiment included a treatment of functional diversity of soil macrofauna communities (LowFD *vs.* HighFD), factorially crossed with a drought treatment (Control with typical summer drought *vs.* Drought including a more severe and prolonged summer drought), with four replicated mesocosms per treatment combination (2 levels of functional diversity of fauna × 2 levels of summer drought × 4 replicates = 16 mesocosms; Supplementary Table 1). Each mesocosm unit consisted of a belowground compartment containing a 1 m^3^ stainless steel lysimeter with a 1 m^2^ surface and a 3.9 m^3^ aboveground compartment enclosed by a highly transparent material permeable to light and UV radiation (250 μm thick Teflon-FEP film, DuPont, USA).

The soil was excavated from a Mediterranean old-field (previously a Mediterranean forest) located at Monferrier-sur-Lez (43°40′52.8″N, 3°52′34.3″E) in January 2018, after removing the herbaceous plants. Three soil layers were excavated separately: the first 10 cm (L1), 10–30 cm (L2) and 30–80 cm (L3). Larger rocks were manually removed from each soil layer. The first two layers, L1 and L2 were then defaunated by gamma irradiation at Synergy Health Marseille SAS facilities, Chusclan, France. Before and after sterilization, sub-samples of soil were extracted to measure soil chemical properties and microbial communities. Compared to other defaunation methods such as autoclaving, or freezing and thawing cycles, the gamma irradiation offers two advantages: (1) a lesser effect on soil physicochemical properties (Berns et al., 2008) and (2) the possibility of selectively target different organism groups with varying doses. We chose a relatively low dose of irradiation (2.5 kGy) to preferentially eliminate macrofauna species while leaving at least part of the microbial community undamaged (McNamara et al., 2003). For sterilization, the soil was bagged in batches of 1 ton each to reach 2.5 kGy at the core of each batch; the peripheral areas received higher doses up to 10 kGy.

The soil was then added by layers to each of the 16 lysimeters, reconstructing the natural layers and bulk density. Suction cups were installed at the bottom of each lysimeter to control water potential. Before adding the soil, we covered the suction cups with a 10 cm layer of gravel, which also facilitated drainage. Layer L3 filled each lysimeter between −90 cm and −30 cm, layer L2 between −30 cm and −10 cm, and finally, layer L1 to the top 10 cm. During the filling of each lysimeter, we added TDR sensors for measurements of volumetric soil moisture content and temperature at three different depths (−60 cm, −30 cm and −10 cm; two sensors per depth).

Each lysimeter was then planted with two individuals of two-year-old saplings of each of four different tree species in March 2018: two evergreen species, *Quercus ilex* L. 1753 and *Arbutus unedo* L. 1753 and two deciduous species, *Quercus pubescens* Willd. 1796 and *Acer monspessulanum* L. 1753. These tree species all co-occur in Mediterranean forests of southern France and differ with regard to their leaf litter quality as well as their mycorrhizal fungi (ectomycorrhiza: the two-oak species, arbuscular mycorrhiza: *A. monspessulanum*, and *A. unedo*). Before planting, the root systems of each sapling were carefully washed to remove soil. The saplings were planted equidistantly spaced to allow similar light exposure. Due to high mortality of *Q. ilex* before the beginning of the experiment, new saplings grown from acorns collected at the Puéchabon forest were planted in February 2019.

We then reconstructed the litter layer in each of the 16 mesocosms in July 2018. The litter layer was composed of equal amounts of all four tree species with a total quantity of 62 ± 0.1 g m^−2^. The leaf litter was collected in surrounding natural forests in fall 2017 (for the two deciduous species) and in June 2018 (for the two evergreen species) and dried at 60°C for 72 h to kill all invertebrates. We later added a layer of manually fragmented leaf litter from all four tree species between the soil surface and the litter layer consisting of intact leaves in June 2019, to improve habitat conditions for the treatment-specific soil fauna (see below).

To ensure that the soil and litter layer contained a microbial and microfauna community representative of the targeted model system (a Mediterranean forest ecosystem), we added an inoculum to each mesocosm created from soils of two forest sites: Montarnaud-France (March 2018) and Puéchabon, France (December 2018). The inoculum was extracted from the top 10 cm of natural forest soil including three of the target species *Q. ilex*, *Q. pubescens* and *A. unedo*. We mixed one volume of soil to five volumes of deionized water, manually blended for 30 min. The solution was passed through a 68 μm sieve, and 5 L per mesocosm were homogeneously added. One week after the second inoculation, sub-samples of soil were extracted to measure microbial communities in the “initial conditions.”

### Climate conditions and the drought treatment

The simulated climate conditions, with the exception of the drought treatment, were identical across all mesocosms during the whole experiment and were based on climatic records from 2013 at the nearby experimental site of Puéchabon, a well-studied Mediterranean forest (Lempereur et al., 2015). The year 2013 was selected as it represented an average year for temperature and precipitation during the 2003–2017 period.

During the summer drought period, the mesocosm watering regime deviated from the 2013 Puéchabon precipitation scenario due to the need to determine the appropriate reduction level for inducing realistic water stress under controlled conditions. A tensiometer-guided approach was employed, regulating drought cycles based on soil water potential at 30 cm depth. These cycles were repeated three times annually from early summer to autumn. Soil water potential was monitored in a reference mesocosm per drought level using a full-range tensiometer (FR T15D, UGT, Germany) to define drought onset and duration.

In 2019, a milder Drought treatment was applied to accommodate sapling root establishment. The Control treatment simulated Mediterranean summer drought, with drought cycles spanning the decline of soil water potential from −0.2 MPa to −1 MPa (moderate to severe water stress). Upon reaching −1 MPa, all eight Control mesocosms were rewatered with the cumulative water lost via evapotranspiration, measured by lysimeter weight changes. In the Drought treatment, drought duration was extended by 30% in the first two cycles and doubled in the third cycle, causing a temporary asynchrony between treatments, with the Control mesocosms receiving water earlier than the Drought mesocosms after each cycle. Consequently, Drought mesocosms received 23% less water than Control between June and October 2019.

In 2020, a similar approach was applied, but water stress in the Drought treatment was intensified by extending drought duration by 30% and reducing precipitation input by 30% per cycle relative to Control. The threshold for defining the drought period was lowered, with soil water potential reaching −1.5 MPa (permanent wilting point) before rewatering. These adjustments resulted in a 38% reduction in precipitation for the Drought treatment between June and October 2020. In both years, following three drought cycles, Control and Drought mesocosms were rewatered according to the Puéchabon precipitation scenario (Supplementary Table 2).

**Figure 1:**
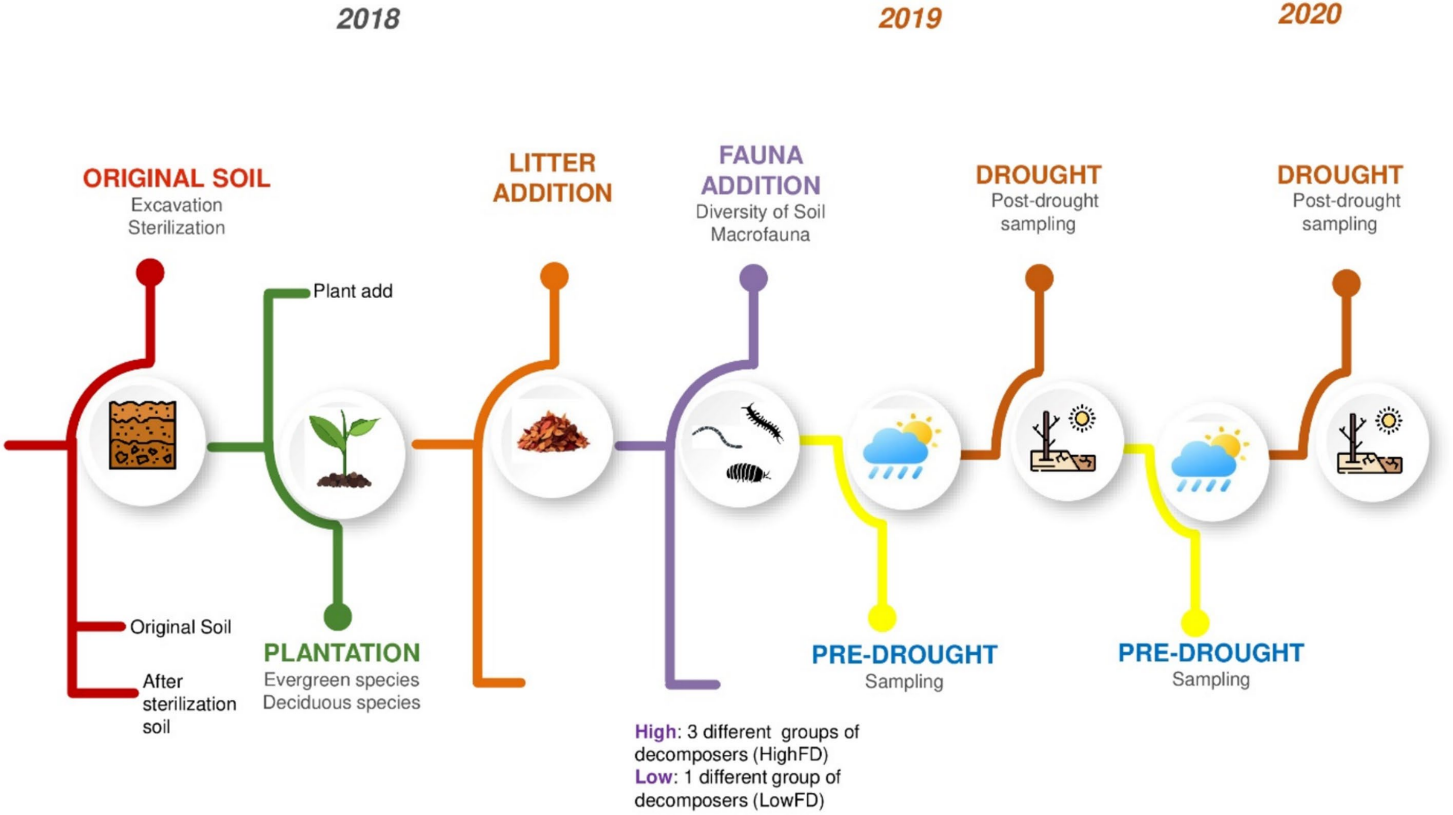
Mesocosm experimental setup. The sampling time correspond to “Pre-drought” (June 2019/2020), and “Post-drought” (September/October 2019/2020), represent samples collected after the three summer drought cycles.

We took soil samples before the drought cycles (“pre-drought” in June of each year, 2019 and 2020) and at the end of the three drought cycles (“post-drought” in September/October of each year) (Figure 1). For each sampling date, three soil cores were randomly collected from the L1 layer (i.e., top soil down to 10 cm) of each mesocosm. The soil was homogenized by sieving through a 4 mm mesh. Three subsamples of 10 g for each mesocosms were taken from the sieved bulk soil and frozen at −80°C for DNA analysis.

### Soil fauna functional diversity

We manipulated soil macrofauna species composition to create two levels of functional diversity, low functional diversity (LowFD) *vs.* high functional diversity (HighFD), while keeping species richness constant at three species per mesocosm. Twelve sapro-geophageous species common in Mediterranean forest were used: three isopod species, *Armadillidium vulgare* Latreille, 1804 (Armadillidae), *Armadillo officinalis* Duméril, 1816 (Armadillidae), *Porcellio laevis* Latreille, 1804 (Porcellionidae); three millipedes (Myriapoda: Diplopoda) *Glomeris marginata* Villers, 1789 (Glomeridae), *Cylindroiulus caeruleocinctus* Wood, 1864 (Julidae), and *Ommatoiulus sabulosus* (Linnaeus, 1758) (Julidae); three endogeic earthworms (Annelida: Lumbricidae) *Aporrectodea caliginosa* (Savigny, 1826), *Aporrectodea icterica* (Savigny, 1826), and *Allolobophora chlorotica* (Savigny, 1826); and three anecic or epianecic earthworms *Aporrectodea nocturna* (Evans, 1946), *Lumbricus terrestris* (Linnaeus. 1758), and *Scherotheca gigas* (Dugès, 1828) (Supplementary Table 1). Although the functional trait-based approaches have received a growing interest in soil fauna ecology (Hedde et al., 2022), the choice of relevant traits explaining the effects of soil fauna species on ecosystem carbon (C) fluxes is still challenging, especially when species belong to different clades. Instead, we have used a hybrid classification mixing phylogenetic (woodlice, millipeds, earthworms) and morphoecological groups (anecic or epianecic *vs.* endogeic). The LowFD communities were composed of three species of the same group, either woodlice, millipede, endogeic or anecic whereas HighFD communities were composed of three species belonging to three different groups. The first fauna addition took place in December 2018, with 4 g fresh weight (FW) per arthropod species and 13 g FW per earthworm species. Subsequent additions of identical biomass were made each year to maintain soil fauna biomass.

### DNA extraction and sequencing

The molecular procedure was in accordance with Prada-Salcedo et al. (2022). The DNA extraction and sequencing process were conducted on each soil sample collected from every mesocosm unit. Briefly, the microbial DNA was isolated from 400 mg of each soil sample using a Power Soil DNA Isolation Kit (Qiagen Laboratories Inc., Solana Beach, USA) following the manufacturer’s instructions. After collecting DNA, it was quantified using NanoDrop equipment (Thermo Fisher Scientific, Germany). The V4 region of the 16S rRNA gene was amplified using the primers P5_8N_515F and P5_7N_515F together with P7_2N_806R and P7_1N_806R (Caporaso et al., 2011; Moll et al., 2018). PCR amplification was performed using 7.50 μL of KAPA HiFi HotStart ReadyMix DNA Polymerase (Kapa, Roche or Sigma) and 0.3 μL of each primer with 2 μL of template in 15 μL reaction. The amplification program was initiated at 95°C for 3 min, followed by 30 cycles of 95°C for 50 s, 55°C for 50 s and 72°C for 60 s, with a final extension of 72°C for 7 min. PCR products were purified using AMPure XP beads (Beckman Coulter, Krefeld, Germany). Indexing and sequencing were performed using Nextera XT Illumina Index Kit (Illumina) according to the manufacturer’s instructions. Library quantification was done following Prada-Salcedo et al. (2022) Illumina MiSeq sequencing was performed at the Department of Soil Ecology, UFZ-Helmholtz Centre for Environmental Research in Halle (Saale), Germany.

### Bioinformatics and statistical methods

For subsequent bacterial and archaeal data analyses, the “dadasnake” pipeline was used (Weißbecker et al., 2020), which is based on the DADA2 algorithm (Callahan et al., 2016). This pipeline produces amplicon sequence variants (ASVs). The following parameters were used: minimum read length 170 bp (forward and reverse) for read sequences; maximum expected error after truncation was 0.2. Taxonomic assignment was done using the SILVA database v138.1 (Quast et al., 2013). The statistical analyses were performed with R v.4.2.1. “Phyloseq” (McMurdie and Holmes, 2013) and “vegan” (Oksanen et al., 2014) were the main R-packages used to analyze the retrieved 16S amplicon data. Samples were rarefied at the cutoff of 32,000 reads. The resulting 16,712 ASVs were used to evaluate the microbial communities in this experiment and to predict microbial functional abundances using PICRUSt2 (Douglas et al., 2020) with the “per_sequence_contrib” option. This option predicted the functional gene abundances from 16S rRNA gene sequences and linked them to enzyme groups and pathways from the MetaCyc database.^2^ Linear mixed-effects models (LMM) were used to evaluate differences in relative abundances at the phylum level and microbial diversity indices across treatments and time. The effects of factors on these variables were assessed, and comparisons between treatments and time points were estimated using the “emmeans” R package. The microbial community composition was evaluated with the “vegdist” function using Bray–Curtis dissimilarity and visualized by non-metric multidimensional scaling (NMDS). The impact of soil macrofauna functional diversity on soil microbial communities was tested by permutational multivariate analysis of variance (PERMANOVA). The “indicspecies” (package version 1.7.14) was used to identify microbial indicator taxa associated with drought and macrofauna functional diversity for time points: Prolonged drought and Prolonged Intense drought, because these were the time points to investigate the microbial communities under post-drought conditions. The selected species indicator taxa were tested for differential abundances and represented with heat trees with the “metacoder” (Foster et al., 2017) applying a Wilcoxon Rank Sum test and represented with a log of ratio of median abundances (*p* value = 0.05).

The results of the predicted functional gene abundances allowed the selection of relevant enzymes associated with abiotic stress for each ASV. This process was determined under two criteria: (1) genes had a relative abundance function between 0.01 and 100, and (2) genes were associated with enzymatic groups related to three types of responses to drought abiotic stress, such as oxidative stress, synthesis of the cell wall, and gene regulation (Table 1). Enzyme abundances were associated with their corresponding taxa at the phylum level and statistically differences analyzed using the Wilcoxon Rank Sum test (*p* value = 0.05).

**Table 1:** Enzyme groups involved in microbial regulation and response to drought abiotic stress in soil.

Abiotic stress	Enzymatic group (Function by MetaCyc)	Common name	Description related to ecological roles	References
Oxidative Stress	EC:1.11.1.6	Catalase	Exposure to abiotic stresses such as temperature extremes, drought, salinity, and nutrient deficiency causes an increase in the production of reactive oxygen species (ROS). Part of the defense mechanisms against toxic damage from these compounds are enzymatic antioxidants. To control ROS homeostasis, cells possess certain antioxidants systems, which include glutathione and ascorbate peroxidases, superoxide dismutase and catalase	Bogati and Walczak (2022), Grover et al. (2011), Omae and Tsuda (2022) and Solana García Andrea (2021)
	EC:1.15.1.1	Superoxide dismutase		
	EC:2.5.1.18	Glutathione transferase		
	EC:1.8.1.7	Glutathione-disulfide reductase		
	EC:7.1.1.9	Cytochrome-c oxidase		
Synthesis cell wall, DNA Damage	EC:2.7.11.1	Serine–Threonine kinase (STPKs)	In bacteria, STPKs determine cell shape, cell wall biosynthesis and remodeling of peptidoglycan layer, morphogenesis, cell division, chromosome segregation, and developmental processes like sporulation and germination spores	Shemesh and Chaia (2013) and Rajpurohit et al. (2022)
	EC:2.7.13.3	Histidine kinase	Promotes biofilm formation	
Regulation response to abiotic stress	EC:5.2.1.8	Peptidylprolyl isomerase (Cyclophilins)	Catalyze the folding of target proteins, also participate in adaptation to environmental stress, cell cycle control and transcriptional regulation	Kim et al. (2010) and Roset et al. (2013)

Estimation of enzyme groups was performed based on their relative abundance and their functions associated with the regulation and response of microbial communities to drought stress conditions, including oxidative stress, DNA damage, and regulation of abiotic stress.

## Results

### Microbial abundance and enriched taxa

**Figure 2:**
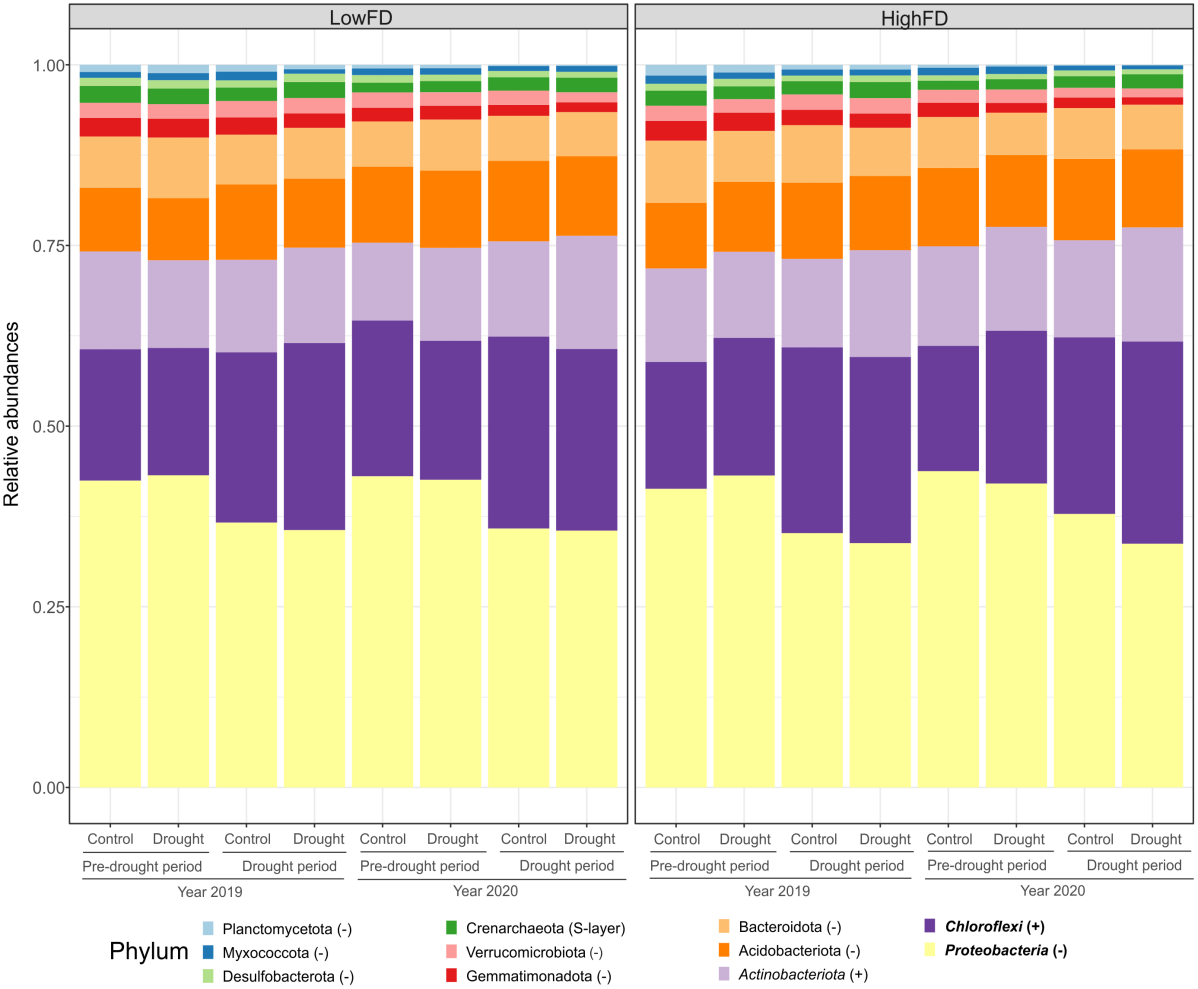
Relative abundances of top 100 abundant bacterial and archaeal ASVs at phyla level in low (LowFD) and high (HighFD) macrofauna functional diversity treatments. Each bar represents a mesocosms units under either Control or Drought conditions. Data are grouped by sampling time and year: ‘Pre-drought’ (samples collected before drought treatment, representing the Puéchabon climate) and ‘Post-drought’ (samples collected after the drought period) for 2019 and 2020. Phyla in bold font show significant differences in relative abundance across each sampling period and within each functional diversity treatment. Phyla in italic indicate significant differences in the relative abundances between the HighFD and LowFD macrofauna functional diversity levels. Statistical details according to Linear Mixed Models (LMM) are provided in Supplementary Tables 3, 4.

Our results indicate that the low dose of gamma irradiation applied after the soil was excavated in 2018 had an effect on microbial species but allowed the majority of the microbial community to persist. Moreover, later in the experiment, after the mesocosms had been established, planted with trees, and supplemented with a litter layer, soil macrofauna, and two inoculations of soil slurry from natural forests, the number of reads and species diversity increased steadily during 2019 and 2020 (Supplementary Figure S1). The continuously changing microbial community over time may also suggest a relatively long establishment phase for microbial communities. We used ASVs from the experimental years 2019 and 2020, to evaluate microbial communities and predict functional gene abundances as a function of the combined treatments of macrofauna functional diversity and drought. The relative abundances at the phylum level throughout the two experimental years for high functional diversity (HighFD) and low functional diversity (LowFD) of macrofauna diversity levels are presented in Figure 2. The results indicate a significant effect of sampling periods, i.e., pre-drought *vs.* drought periods, on microbial relative abundances (*F* = 3.29, *p* < 0.01). We observed a general decrease in relative abundances from 2019 to 2020 for some phyla, such as Gemmatimonadota, Verrucomicrobiota and Planctomycetota, for both macrofauna functional diversity levels. Significant differences across sampling periods, specifically between pre-drought and drought periods, were observed in Proteobacteria and Chloroflexi, which also showed the highest relative abundances. Proteobacteria decreased in abundance during the drought period for both Control and Drought treatments. Conversely, Chloroflexi increased in abundance during drought periods (Figure 2 and Supplementary Table 3).

Overall relative abundances at the phylum level did not differ between high and low macrofauna functional diversity (*F* = 2.11, *p* = 0.14). However, the results indicate a significant effect of the drought treatments on the relative abundances (*F* = 4.99, *p* = 0.02) and an interaction effect between drought treatments and macrofauna functional diversity levels (*F* = 3.95, *p* = 0.04). For example, Gram-positive bacteria, such as Chloroflexi and Actinobacteriota, exhibited higher relative abundances under HighFD conditions during drought (in both years 2019 and 2020) and even during the pre-drought period between the 2019 and 2020 summer droughts compared to the LowFD treatment (Figure 2 and Supplementary Table 4).

**Figure 3:**
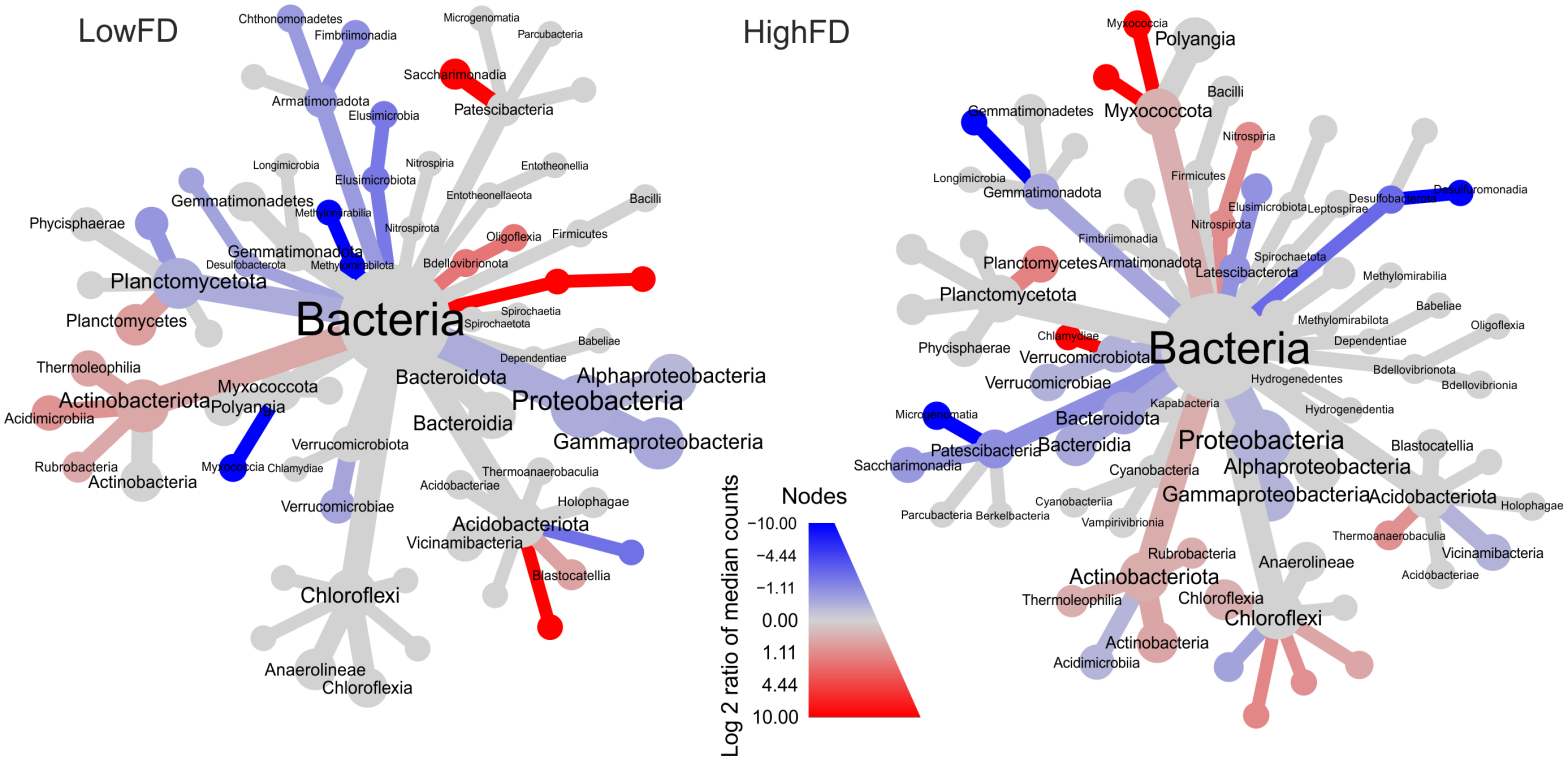
Microbial differential abundances under low (LowFD) and high (HighFD) macrofauna functional diversity levels. Only phyla associated with drought periods (September–October 2019 and October 2020) under Control and Drought treatments are presented. Taxonomy is presented until order level. Significant differences abundances are presented between the Control treatment (blue) and the Drought treatment (red). Taxa that do not respond are shown in gray. Branches without labels represent undefined taxa.

Our species indicator analysis for drought periods revealed that from a total of 16,712 ASVs, 130 and 158 ASVs were exclusive to the control treatment for LowFD and HighFD, respectively. In the drought treatments, a total of 173 and 167 ASVs were exclusive for LowFD and HighFD, respectively (across both years 2019 and 2020, Supplementary Figure S2-Venn diagram). Overall, we found differential abundances among taxa during the drought periods of both years (2019 and 2020) between LowFD and HighFD (Figure 3). A consistent trend for both fauna functional diversity levels was that Actinobacteriota was related to drought treatments (2019 and 2020), and at the class level within this phylum, the Thermoleophilia and Rubrobacteria were more abundant under drought than in the control treatments. A similar case was observed for the Planctomycetes, where these taxa were abundant under drought treatments. In an opposite direction, and also consistent between macrofauna functional diversity levels, the Desulfobacterota phylum showed higher abundances in drought than in control treatments (Figure 3). Other class such as Acidimicrobiia, Saccharimonadia, or Myxoccocia showed opposing responses in abundance according to the functional diversity of soil fauna. The differential abundance analysis also revealed differential taxa responding to the control and drought treatments for HighFD and LowFD. In the HighFD, 78 ASVs were detected with significantly higher differential abundances under control conditions, while 87 ASVs exhibited significantly higher differential abundances under drought conditions. For the LowFD samples, 88 ASVs were associated with significantly higher differential abundances in the control treatment, whereas 65 ASVs displayed significantly higher differential abundances under drought conditions (Supplementary Tables 5, 6).

### Diversity and microbial community composition

**Figure 4:**
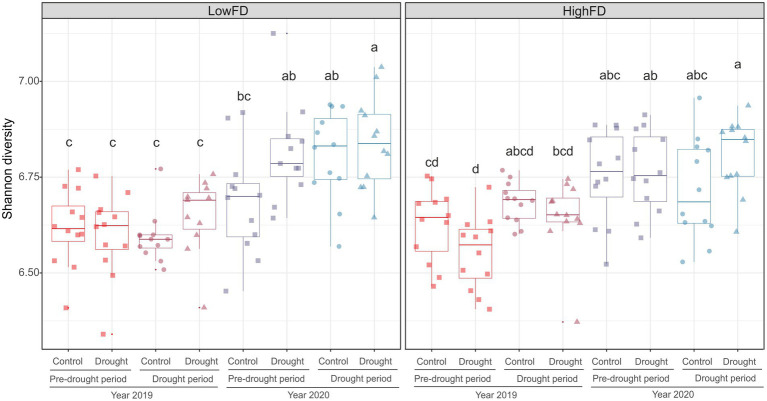
Microbial Shannon diversity in low (LowFD) and high (HighFD) macrofauna functional diversity levels. Each boxplot represents the data from four replicates per treatment combination and time of sampling. Data are arranged along progressive experimental duration from early summer 2019 (before the first summer drought) to autumn 2020 (after the second summer drought). Different letters above boxplots indicate significant diversity differences between treatments (Control vs. Drought) and among sampling dates, as determined by Linear Mixed Models (LMM). Statistical details are provided in Supplementary Tables 7, 8.

The microbial Shannon diversity was comparable between HighFD and LowFD treatments (Figure 4). Likewise, the Drought treatment had no impact on microbial diversity. However, Shannon diversity generally increased over time for both levels of macrofauna functional diversity (*F* = 16.44, *p* < 0.01). The differences over time appeared more pronounced for LowFD than for HighFD treatments. For instance, the Shannon diversity of microbial communities from the HighFD treatment exposed to drought was comparable between 2019 and 2020. Specifically, microbial diversity in the Drought treatment in 2019 was similar to that in the pre-drought period of 2020, and this pre-drought diversity in 2020 was comparable to the Drought treatment diversity in the same year (Figure 4). In contrast, the microbial Shannon diversity of communities from the LowFD treatment differed considerably among measurements over time for the same years, and Drought treatments were less comparable over time (Figure 4 and Supplementary Tables 7, 8).

**Figure 5:**
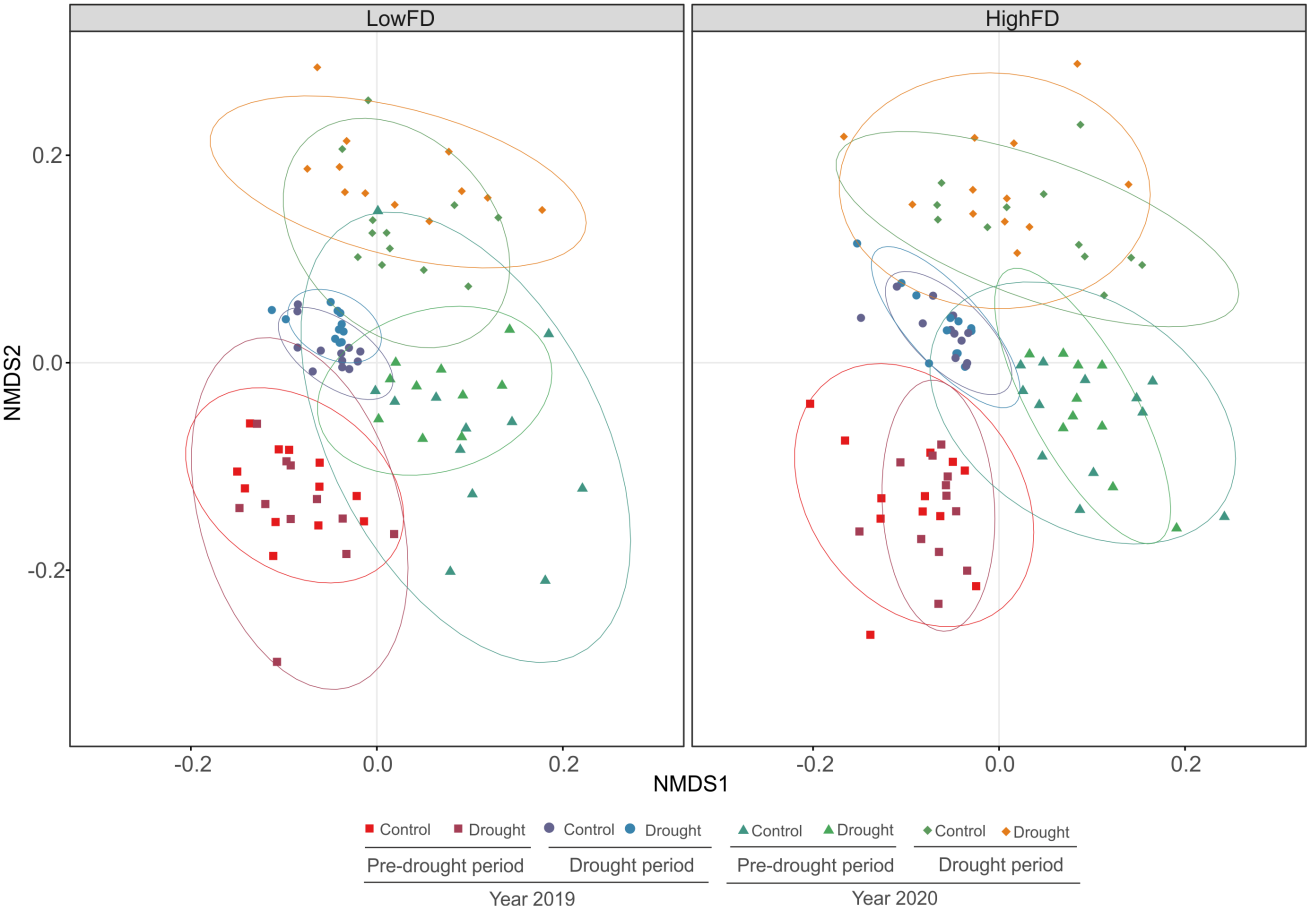
Microbial community composition in low (LowFD) and high (HighFD) macrofauna functional diversity levels. Each color represents samples grouped by sampling date: ‘pre-drought’ (samples collected in early summer before the drought cycles started) and ‘post-drought’ (samples collected immediately after the three drought cycles during summer), under either Control or Drought conditions.

Our analysis revealed significant differences in microbial community composition. The PERMANOVA results showed a slight but significant effect of macrofauna functional diversity (*R*^2^ = 0.006, *F* = 1.51, *p* = 0.03), and stronger effects were detected among sampling time periods (*R*^2^ = 0.16, *F* = 12.65, *p* < 0.01). Moreover, the results indicate an overall Drought treatment significant effect (*R*^2^ = 0.008, *F* = 2.00, *p* < 0.01). This Drought treatment effect was more pronounced under prolonged and more severe summer drought, showing that HighFD exhibited a weaker difference of the Control Vs. Drought treatment (*R*^2^ = 0.034, *F* = 1.64, *p* < 0.009), for the years 2019 and 2020, compared to the same treatments with LowFD (*R*^2^ = 0.037, *F* = 1.77, *p* < 0.01). NMDS analysis showed similar differences among sampling dates regardless of the macrofauna functional diversity treatment (Figure 5). The first axis primarily separated the data according to time of sampling, with 2019 samples to the left and 2020 samples to the right. Likewise, microbial communities were also mainly separated with ongoing experimental duration, and thus, drought impact along the second axis of the NMDS plot. This resulted in microbial communities shifting from the bottom left to the top right of the NMDS plot in their composition over time, with some differences between Control and Drought.

### Estimated enzyme responses associated with drought

**Figure 6:**
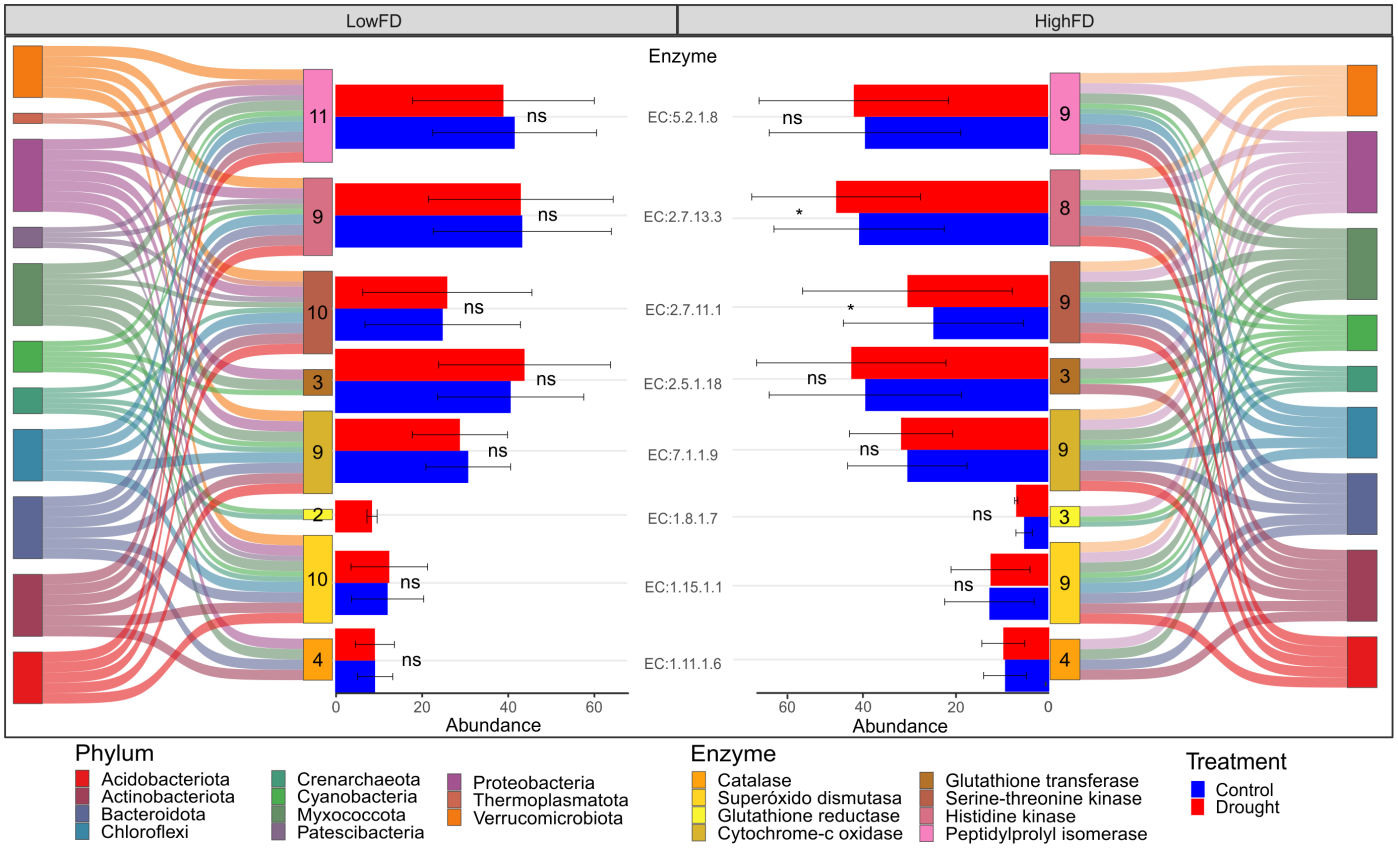
Estimated enzyme abundances in LowFD and HighFD macrofauna treatments and their associated microbial taxa at the phylum level. Only microbial enzyme measurements estimated after the completion of the drought cycles autumn are included, with data from 2019 and 2020 combined. Error bars represent standard deviation. Numbers within enzyme boxes indicate the number of microbial taxa at phylum level supporting the enzyme abundances.

The estimated abundance of genes coding for microbial enzymes involved in drought response is shown in Figure 6. Overall, the results suggest little impact of prolonged and more severe summer drought on the abundance of drought stress-related enzymes. There were two exceptions, both in the HighFD treatment, with Crenarchaeota and Proteobacteria showing higher enzyme abundances under the Drought condition, compared to the Control (Figure 6). This effect was significant for the histidine kinase and serine–threonine kinase (Supplementary Table 9). Interestingly, there were eleven phyla associated with the expression of drought stress-related enzymes under the LowFD, compared to only nine phyla associated with the HighFD. The Patescibacteria and Thermoplasmatota (Archaea) were phyla present in the LowFD, but absent in the HighFD. These findings suggest that the enzyme abundances were supported by a greater number of taxa under LowFD than under HighFD.

## Discussion

In this study, we investigated the impact of repeated drought events during two consecutive years on the diversity and composition of soil prokaryotic communities, and explored whether these effects were influenced by the functional diversity of soil fauna as well as prolonged and more severe summer droughts. Our results showed that soil microbial communities were continuously evolving from a common starting point at the beginning of the experiment over the two experimental years. This temporal shift in microbial community composition was partly affected by the combined treatments of prolonged and more severe summer drought and distinct functional diversity of soil macrofauna. Microbial diversity generally increased during the experiment, reaching the highest diversity in 2020 for both macrofauna functional diversity treatments. Most of this increase in microbial diversity and change in community composition appeared to be related to inherent successional dynamics after the setup of the mesocosm, followed by an establishment phase (Thakur and Geisen, 2019).

For the years 2019 and 2020, our results revealed changes in the relative abundances of soil microbes exposed to a series of drought cycles each summer. At the phylum level of the dominant microbial taxa, we observed a clear shift between Proteobacteria and Chloroflexi, with the latter being less affected by drought conditions. The Gram-positive phylum Chloroflexi has been reported previously to be drought tolerant (Santos-Medellín et al., 2017). In contrast, the Gram-negative Proteobacteria were highly responsive to drought, but their responses may vary depending on specific classes involved and as a function of plant responses. For example, the Alphaproteobacteria often show an increased abundance following drought, while Betaproteobacteria tend to respond negatively to drought stress (Fan et al., 2023; Moreno-Espíndola et al., 2018). The species indicator analysis allows differentiation of relevant microbial taxa as a function of treatment-specific responses and revealed here eleven taxa that were particularly sensitive to the drought treatment, regardless of the macrofauna functional diversity treatment. Some of them have been identified before as drought-sensitive taxa, such as Actinobacteriota, Acidobacteriota, Proteobacteriota, Planctomycetota, Myxococcota, Chloroflexi and Gemmatimonadota (Bogati and Walczak, 2022; Chodak et al., 2015; Maisnam et al., 2023). Gram-positive bacteria often possess drought-resistant traits, allowing some microorganisms to recover rapidly from drought stress. Gram-negative bacteria, though more drought-sensitive, compensate through fast reproduction after the drought has passed and they can attain initial abundances rather quickly (Watzinger et al., 2023).

The differential abundance analysis revealed differences between Control and Drought conditions. The Thermoleophilia and Rubrobacteria were particularly abundant under the prolonged and more severe drought cycles in 2020. These groups are often found in arid environments, and Rubrobacteria have the capacity to produce stress-resistant spores and osmoprotectants, and can also form biofilms, potentially helping plants to cope with drought stress during harsh summer conditions (Narsing Rao et al., 2022). In contrast, our results show that the ASVs related to the phylum Desulfobacterota, which thrive in water-saturated environments, were negatively affected by the Drought treatment (Munyai et al., 2021). These findings support our first hypothesis that Gram-positive communities are favored under drought conditions. In general, severe drought requires microorganisms to adopt strategies to enhance their resilience. For example, they may create resistance structures, such as increasing peptidoglycan content in their cell walls or accumulating osmolytes to overcome water stress. Additionally, soil microbes may accumulate carbon (C) compounds to adapt to the reduced accessibility of organic substrates or develop structures to resist desiccation. Many of these adaptive mechanisms are primarily developed by Gram-positive bacteria. Consequently, it is believed that Gram-negative bacteria are more sensitive to abiotic stress (Marañón-Jiménez et al., 2022; Moreno-Espíndola et al., 2018).

We expected that higher functional diversity of soil macrofauna would increase microbial diversity. In contrast to this initial hypothesis, Shannon diversity was similar between HighFD and LowFD conditions. This suggests that, at least within the time span of this experiment, the effects of higher soil macrofauna functional diversity did not lead to measurable changes in microbial diversity. Nevertheless, the results from two experimental years indicated that HighFD led to less fluctuation in microbial communities over time. This buffering effect is supported by the observation that Shannon microbial diversity in HighFD varied less across time compared to LowFD. The more comparable diversity observed during the drought period of 2019 and the more intense drought in 2020 for HighFD treatments suggests a legacy effect in the soil. This legacy effect is reflected in the comparable microbial diversity of the Drought treatment in Pre-drought samples (2020) with the previous and subsequent sampled periods. Notably, this effect was not observed in the LowFD treatments. Therefore, it is possible that over an extended experimental period, the dynamic balance of macrofauna-microbe interactions would intensify, creating more complex interactions to face drought. The potential stability in microbial diversity under HighFD conditions could be attributed to several non-exclusive factors, such as an enhanced soil structure and microhabitat diversity created by a functionally more diverse macrofauna (Meyer et al., 2021; Peguero et al., 2021; Scheu et al., 2005), a generally more stable soil community due to more complex food web interactions and potentially beneficial feedback effects from a functionally more diverse macrofauna (Guidi et al., 2022; Olayemi et al., 2022). Collectively, these different factors may contribute to a more stable and resilient microbial community under HighFD conditions, potentially explaining the observed legacy effect and the reduced variability in diversity over time.

According to the NMDS and PERMANOVA results, macrofauna functional diversity, prolonged and more severe drought, and sampling time all influenced microbial community composition. The strongest effects were attributed to time, with comparatively less influence of macrofauna functional diversity and Drought. The divergent microbial community composition between HighFD and LowFD macrofauna communities under drought could lead to altered microbial activities, with potential consequences for organic matter decomposition and nutrient cycling (Malik and Bouskill, 2022). We found a weaker impact of the Drought treatment on microbial composition under HighFD, suggesting that after the multiple drought cycles, microbial communities could be more adapted to stress and become more resilient. Moreover, this community under HighFD was associated with a Gram-positive microbial community. The microbial community under HighFD treatments may mitigate drought impacts more effectively than those under LowFD conditions, potentially due to faunal diversity-induced processes such as improved soil aeration, enhanced water infiltration, more efficient mixing of organic matter into deeper soil layers, increased formation of soil aggregates, and greater fragmentation of plant litter. These processes accelerate litter decomposition and create diverse microhabitats for microbial colonization by taxa with more drought-adapted traits (Olayemi et al., 2022).

The estimates of stress response-related enzyme activities allowed us to evaluate potential microbial responses to oxidative stress, DNA damage, and regulatory response to abiotic stress. These results suggest a higher relative gene abundance of ASVs for eight enzymes associated with prolonged and more severe drought compared to the control. In general, the abundance of genes associated with enzymes having antioxidant activity appears to be lower compared to those related to DNA damage repair and abiotic stress regulation. Despite their low abundance, these antioxidant enzymes are critical for microbial responses to drought. However, our results showed a greater abundance of genes associated with cyclophilins. Cyclophilins are a family of proteins that play important roles in drought stress responses in plants. Previous studies have demonstrated that cyclophilin expression changes in response to abiotic stress in both plants and microorganisms (Kim et al., 2010; Olejnik et al., 2021).

Significant differences in the abundance of serine–threonine kinase and histidine kinase enzymes were observed between the Control and Drought treatments. These differences were particularly pronounced under HighFD of the macrofauna, supporting our third hypothesis. Both enzymes are involved in the response to abiotic stress, providing protection against DNA damage and facilitating other adaptation mechanisms to desiccation and high temperatures (Janczarek et al., 2018; Pereira et al., 2011). These kinases often work in concert within complex signaling networks to regulate cellular responses to stress. Such responses include production of biofilms, cell wall modifications, and sporulation, which are the primary strategies employed by microorganisms to survive extreme drought conditions (Janczarek et al., 2018; Pereira et al., 2011; Rajpurohit et al., 2022; Shemesh and Chaia, 2013). It is interesting to note that the expression of these enzymes under HighFD is carried out by a smaller number of phyla than in LowFD, but by taxa more specialized in withstanding dry conditions. This could be related to the current knowledge that diverse drought-tolerant communities may also have a larger capacity to face abiotic stress than a higher abundance of non-specialized taxa (Metze et al., 2023). A higher macrofauna functional diversity may be associated with more diverse interactions with microbial communities better adapted to drought periods (Peguero et al., 2021). Therefore, the synergistic interactions between different species in the microbial community and soil fauna community may improve the functional capacity of the ecosystem to face drought stress periods (Yang et al., 2021).

## Conclusion

The main findings of our study reveal that Gram-positive bacterial communities play a central role in coping with prolonged and more severe drought, demonstrating an adaptive shift toward taxa with drought-tolerant traits. Additionally, higher macrofauna functional diversity appears to stimulate the abundance of specific microbial groups that are better adapted to drought. Greater macrofauna functional diversity may also contribute to more stable microbial communities over time, particularly during drought periods, as reflected in the more similar community composition observed in the HighFD treatment when comparing Control and Drought treatments. Furthermore, the drought-tolerant bacterial taxa identified in this study were enriched with genes associated with drought resilience, and more so in the HighFD condition (for histidine kinase and glutathione transferase), which may enhance their capacity to withstand more severe droughts predicted with ongoing climate change. These findings underscore the critical role of macrofauna-microbial interactions in maintaining soil ecosystem functionality and buffering adverse environmental conditions, highlighting adaptive shifts in microbial community structure and the enrichment of drought-tolerant traits as key mechanisms for resilience.

## Data Availability

The datasets presented in this study can be found in online repositories. The names of the repository/repositories and accession number(s) can be found below: https://www.ebi.ac.uk/ena, PRJEB85576 and experiment accession ERX13652766, ERR14251927.

## Footnotes

^1^ https://www.ecotron.cnrs.fr/en/mesocosms/

^2^ https://metacyc.org/

## References

[ref1] Abdelaal K. Alkahtani M. Attia K. Hafez Y. Király L. Künstler A. (2021). The role of plant growth-promoting bacteria in alleviating the adverse effects of drought on plants. Biology 10:520. doi: 10.3390/biology10060520, PMID: 34207963 PMC8230635

[ref2] Berns A. E. Philipp H. Narres H. D. Burauel P. Vereecken H. Tappe W. (2008). Effect of gamma-sterilization and autoclaving on soil organic matter structure as studied by solid state NMR, UV and fluorescence spectroscopy. Eur. J. Soil Sci. 59, 540–550. doi: 10.1111/j.1365-2389.2008.01016.x

[ref3] Bhaduri D. Sihi D. Bhowmik A. Verma B. C. Munda S. Dari B.. (2022). A review on efective soil health bio-indicators for ecosystem restoration and sustainability 13:938481. doi: 10.3389/fmicb.2022.938481 PMC9428492 36060788

[ref4] Bogati K. Walczak M. (2022). The impact of drought stress on soil microbial community, enzyme activities and plants. Agronomy 12:189. doi: 10.3390/agronomy12010189

[ref5] Callahan B. J. McMurdie P. J. Rosen M. J. Han A. W. Johnson A. J. A. Holmes S. P. (2016). DADA2: high-resolution sample inference from Illumina amplicon data. Nat. Methods 13, 581–583. doi: 10.1038/nmeth.3869, PMID: 27214047 PMC4927377

[ref6] Caporaso J. G. Lauber C. L. Walters W. A. Berg-Lyons D. Lozupone C. A. Turnbaugh P. J.. (2011). Global patterns of 16S rRNA diversity at a depth of millions of sequences per sample. Proc. Natl. Acad. Sci. USA 108, 4516–4522. doi: 10.1073/pnas.1000080107, PMID: 20534432 PMC3063599

[ref7] Chamorro-Martínez Y. Torregroza-Espinosa A. Pallares M. Osorio D. Paternina A. Echeverría-González A. (2022). Soil macrofauna, mesofauna and microfauna and their relationship with soil quality in agricultural areas in northern Colombia: ecological implications. Rev. Bras. Ciênc. Solo 46:e20210132. doi: 10.36783/18069657rbcs20210132

[ref8] Chodak M. Gołębiewski M. Morawska-Płoskonka J. Kuduk K. Niklińska M. (2015). Soil chemical properties affect the reaction of forest soil bacteria to drought and rewetting stress. Ann. Microbiol. 65, 1627–1637. doi: 10.1007/s13213-014-1002-0, PMID: 26273241 PMC4529456

[ref9] Cole L. Bradford M. A. Shaw P. J. A. Bardgett R. D. (2006). The abundance, richness and functional role of soil meso- and macrofauna in temperate grassland-a case study. Appl. Soil Ecol. 33, 186–198. doi: 10.1016/j.apsoil.2005.11.003

[ref10] Coleman D. C. Geisen S. Wall D. H. (2023). “Soil fauna: occurrence, biodiversity, and roles in ecosystem function” in Soil microbiology, ecology and biochemistry, fifth edition. eds. Paul E. A. Frey S. D. (Amsterdam, Netherlands: Elsevier), 131–159.

[ref11] Coulis M. Fromin N. David J. F. Gavinet J. Clet A. Devidal S.. (2015). Functional dissimilarity across trophic levels as a driver of soil processes in a Mediterranean decomposer system exposed to two moisture levels. Oikos 124, 1304–1316. doi: 10.1111/oik.01917

[ref12] Crowther T. W. Boddy L. Hefin Jones T. (2012). Functional and ecological consequences of saprotrophic fungus-grazer interactions. ISME J. 6, 1992–2001. doi: 10.1038/ismej.2012.53, PMID: 22717883 PMC3475375

[ref13] Douglas G. M. Maffei V. J. Zaneveld J. R. Yurgel S. N. Brown J. R. Taylor C. M.. (2020). PICRUSt2 for prediction of metagenome functions. Nat. Biotechnol. 38, 685–688. doi: 10.1038/s41587-020-0548-6, PMID: 32483366 PMC7365738

[ref14] Fan W. Tang F. Wang J. Dong J. Xing J. Shi F. (2023). Drought-induced recruitment of specific root-associated bacteria enhances adaptation of alfalfa to drought stress. Front. Microbiol. 14:1114400. doi: 10.3389/fmicb.2023.1114400, PMID: 36910228 PMC9995459

[ref15] Foster Z. S. L. Sharpton T. J. Grünwald N. J. (2017). Metacoder: an R package for visualization and manipulation of community taxonomic diversity data. PLoS Comput. Biol. 13:e1005404. doi: 10.1371/journal.pcbi.1005404, PMID: 28222096 PMC5340466

[ref16] Gillespie L. M. Prada-Salcedo L. D. Shihan A. Fromin N. Goldmann K. Milcu A.. (2023). Taxonomical and functional responses of microbial communities from forest soils of differing tree species diversity to drying-rewetting cycles. Pedobiologia 97–98:150875. doi: 10.1016/j.pedobi.2023.150875, PMID: 40415499

[ref17] Grover M. Ali S. Z. Sandhya V. Rasul A. Venkateswarlu B. (2011). Role of microorganisms in adaptation of agriculture crops to abiotic stresses. World J. Microbiol. Biotechnol. 27, 1231–1240. doi: 10.1007/s11274-010-0572-7

[ref18] Guidi C. Frey B. Brunner I. Meusburger K. Vogel M. E. Chen X.. (2022). Soil fauna drives vertical redistribution of soil organic carbon in a long-term irrigated dry pine forest. Glob. Chang. Biol. 28, 3145–3160. doi: 10.1111/gcb.16122, PMID: 35124879 PMC9306871

[ref19] Hedde M. Blight O. Briones M. J. I. Bonfanti J. Brauman A. Brondani M.. (2022). *A* common framework for developing robust soil fauna classifications. Geoderma 426:116073. doi: 10.1016/j.geoderma.2022.116073

[ref20] Heemsbergen D. A. Berg M. P. Loreau M. Van Hal J. R. Faber J. H. Verhoef H. A. (2004). Biodiversity effects on soil processes explained by interspecific functional dissimilarity. Science 306, 1019–1020. doi: 10.1126/science.1101865, PMID: 15528441

[ref21] Hugerth L. W. Andersson A. F. (2017). Analysing microbial community composition through amplicon sequencing: from sampling to hypothesis testing. Front. Microbiol. 8:1561. doi: 10.3389/fmicb.2017.01561 28928718 PMC5591341

[ref22] Ineson P. Anderson J. M. (1985). Aerobically isolated bacteria associated with the gut and faeces of the litter feeding macroarthropods *Onicus asellus* and *Glomeris marginata*. Soil Biol. Biochem. 17, 843–849. doi: 10.1016/0038-0717(85)90145-2

[ref23] Janczarek M. Vinardell J. M. Lipa P. Karaś M. (2018). Hanks-type serine/threonine protein kinases and phosphatases in bacteria: roles in signaling and adaptation to various environments. Int. J. Mol. Sci. 19:2872. doi: 10.3390/ijms19102872, PMID: 30248937 PMC6213207

[ref24] Joly F. X. Coq S. Coulis M. David J. F. Hättenschwiler S. Mueller C. W.. (2020). Detritivore conversion of litter into faeces accelerates organic matter turnover. Commun. Biol. 3:660. doi: 10.1038/s42003-020-01392-4, PMID: 33177652 PMC7658975

[ref25] Kim I. S. Shin S. Y. Kim Y. S. Kim H. Y. Lee D. H. Park K. M.. (2010). Expression of yeast cyclophilin a (Cpr1) provides improved stress tolerance in *Escherichia coli*. J. Microbiol. Biotechnol. 20, 974–977. doi: 10.4014/jmb.0911.11005, PMID: 20622494

[ref26] Lehmann J. Bossio D. A. Kögel-Knabner I. Rillig M. C. (2020). The concept and future prospects of soil health. Nat. Rev. Earth Environ. 1, 544–553. doi: 10.1038/s43017-020-0080-8, PMID: 33015639 PMC7116140

[ref27] Lempereur M. Martin-Stpaul N. K. Damesin C. Joffre R. Ourcival J. M. Rocheteau A.. (2015). Growth duration is a better predictor of stem increment than carbon supply in a Mediterranean oak forest: implications for assessing forest productivity under climate change. New Phytol. 207, 579–590. doi: 10.1111/nph.13400, PMID: 25913661

[ref28] Li K. Veen G. F. ten Hooven F. C. Harvey J. A. van der Putten W. H. (2023). Soil legacy effects of plants and drought on aboveground insects in native and range-expanding plant communities. Ecol. Lett. 26, 37–52. doi: 10.1111/ele.14129 36414536 PMC10098829

[ref29] Luan J. Li S. Liu S. Wang Y. Ding L. Lu H.. (2024). Biodiversity mitigates drought effects in the decomposer system across biomes. Proc. Natl. Acad. Sci. USA 121:e2313334121. doi: 10.1073/pnas.2313334121, PMID: 38498717 PMC10990129

[ref30] Lubbers I. M. Berg M. P. De Deyn G. B. van der Putten W. H. van Groenigen J. W. (2020). Soil fauna diversity increases CO_2_ but suppresses N_2_O emissions from soil. Glob. Chang. Biol. 26, 1886–1898. doi: 10.1111/gcb.14860, PMID: 31587448 PMC7078878

[ref31] Maisnam P. Jeffries T. C. Szejgis J. Bristol D. Singh B. K. Eldridge D. J.. (2023). Severe prolonged drought favours stress-tolerant microbes in Australian drylands. Microb. Ecol. 86, 3097–3110. doi: 10.1007/s00248-023-02303-w, PMID: 37878053 PMC10640424

[ref32] Malik A. A. Bouskill N. J. (2022). Drought impacts on microbial trait distribution and feedback to soil carbon cycling. Funct. Ecol. 36, 1442–1456. doi: 10.1111/1365-2435.14010

[ref33] Marañón-Jiménez S. Asensio D. Sardans J. Zuccarini P. Ogaya R. Mattana S.. (2022). Seasonal drought in Mediterranean soils mainly changes microbial C and N contents whereas chronic drought mainly impairs the capacity of microbes to retain P. Soil Biol. Biochem. 165:108515. doi: 10.1016/j.soilbio.2021.108515

[ref34] Martin P. A. Fisher L. Pérez-Izquierdo L. Biryol C. Guenet B. Luyssaert S.. (2024). Meta-analysis reveals that the effects of precipitation change on soil and litter fauna in forests depend on body size. Glob. Change Biol. 30:e17305. doi: 10.1111/gcb.17305, PMID: 38712651

[ref35] McMurdie P. J. Holmes S. (2013). Phyloseq: an R package for reproducible interactive analysis and graphics of microbiome census data. PLoS One 8:e61217. doi: 10.1371/journal.pone.0061217, PMID: 23630581 PMC3632530

[ref36] McNamara N. P. Black H. I. J. Beresford N. A. Parekh N. R. (2003). Effects of acute gamma irradiation on chemical, physical and biological properties of soils. Appl. Soil Ecol. 24, 117–132. doi: 10.1016/S0929-1393(03)00073-8

[ref37] Medina-Sauza R. M. Álvarez-Jiménez M. Delhal A. Reverchon F. Blouin M. Guerrero-Analco J. A.. (2019). Earthworms building up soil microbiota, a review. Front. Environ. Sci. 7:81. doi: 10.3389/fenvs.2019.00081

[ref38] Metze D. Schnecker J. Canarini A. Fuchslueger L. Koch B. J. Stone B. W.. (2023). Microbial growth under drought is confined to distinct taxa and modified by potential future climate conditions. Nat. Commun. 14:5895. doi: 10.1038/s41467-023-41524-y, PMID: 37736743 PMC10516970

[ref39] Meyer S. Kundel D. Birkhofer K. Fliessbach A. Scheu S. (2021). Soil microarthropods respond differently to simulated drought in organic and conventional farming systems. Ecol. Evol. 11, 10369–10380. doi: 10.1002/ece3.7839, PMID: 34367581 PMC8328414

[ref40] Moll J. Kellner H. Leonhardt S. Stengel E. Dahl A. Bässler C.. (2018). Bacteria inhabiting deadwood of 13 tree species are heterogeneously distributed between sapwood and heartwood. Environ. Microbiol. 20, 3744–3756. doi: 10.1111/1462-2920.14376, PMID: 30109768

[ref41] Moreno-Espíndola I. P. Ferrara-Guerrero M. J. Luna-Guido M. L. Ramírez-Villanueva D. A. De León-Lorenzana A. S. Gómez-Acata S.. (2018). The bacterial community structure and microbial activity in a traditional organic milpa farming system under different soil moisture conditions. Front. Microbiol. 9:2737. doi: 10.3389/fmicb.2018.02737, PMID: 30487784 PMC6246654

[ref42] Munyai R. Ogola H. J. O. Modise D. M. (2021). Microbial community diversity dynamics in acid mine drainage and acid mine drainage-polluted soils: implication on mining water irrigation agricultural sustainability. Front. Sustain. Food Syst. 5:701870. doi: 10.3389/fsufs.2021.701870

[ref43] Narsing Rao M. P. Lohmaneeratana K. Bunyoo C. Thamchaipenet A. (2022). Actinobacteria–plant interactions in alleviating abiotic stress. Plants 11:2976. doi: 10.3390/plants11212976, PMID: 36365429 PMC9658302

[ref44] Naylor D. Coleman-Derr D. (2018). Drought stress and root-associated bacterial communities. Front. Plant Sci. 8:2223. doi: 10.3389/fpls.2017.02223, PMID: 29375600 PMC5767233

[ref45] Oksanen J. Blanchet F. G. Kindt R. Legendre P. Minchin P. R. O’Hara R. B. (2014). Vegan: Community Ecology Package. R Package Version 2.6-0. Available at: http://CRAN.Rproject.org/package=vegan

[ref46] Olayemi O. P. Schneekloth J. P. Wallenstein M. D. Trivedi P. Calderón F. J. Corwin J.. (2022). Soil macrofauna and microbial communities respond in similar ways to management drivers in an irrigated maize system of Colorado (USA). Appl. Soil Ecol. 178:104562. doi: 10.1016/j.apsoil.2022.104562

[ref47] Olejnik P. Mądrzak C. J. Nuc K. (2021). Cyclophilins and their functions in abiotic stress and plant–microbe interactions. Biomolecules 11:1390. doi: 10.3390/biom11091390, PMID: 34572603 PMC8464771

[ref48] Omae N. Tsuda K. (2022). Plant-microbiota interactions in abiotic stress environments. MPMI 35, 511–526. doi: 10.1094/MPMI-11-21-0281-FI 35322689

[ref49] Peguero G. Folch E. Liu L. Ogaya R. Peñuelas J. (2021). Divergent effects of drought and nitrogen deposition on microbial and arthropod soil communities in a Mediterranean forest. Eur. J. Soil Biol. 103:103275. doi: 10.1016/j.ejsobi.2020.103275

[ref50] Peng Y. Peñuelas J. Vesterdal L. Yue K. Peguero G. Fornara D. A.. (2022). Responses of soil fauna communities to the individual and combined effects of multiple global change factors. Ecol. Lett. 25, 1961–1973. doi: 10.1111/ele.14068, PMID: 35875902

[ref51] Pereira S. F. F. Goss L. Dworkin J. (2011). Eukaryote-like serine/threonine kinases and phosphatases in Bacteria. Microbiol. Mol. Biol. Rev. 75, 192–212. doi: 10.1128/mmbr.00042-10, PMID: 21372323 PMC3063355

[ref52] Prada-Salcedo L. D. Prada-Salcedo J. P. Heintz-Buschart A. Buscot F. Goldmann K. (2022). Effects of tree composition and soil depth on structure and functionality of belowground microbial communities in temperate European forests. Front. Microbiol. 13:920618. doi: 10.3389/fmicb.2022.920618, PMID: 35910637 PMC9328770

[ref53] Quast C. Pruesse E. Yilmaz P. Gerken J. Schweer T. Yarza P.. (2013). The SILVA ribosomal RNA gene database project: improved data processing and web-based tools. Nucleic Acids Res. 41, D590–D596. doi: 10.1093/nar/gks1219, PMID: 23193283 PMC3531112

[ref54] Rahman N. S. N. A. Hamid N. W. A. Nadarajah K. (2021). Effects of abiotic stress on soil microbiome. Int. J. Mol. Sci. 22:9036. doi: 10.3390/ijms22169036 34445742 PMC8396473

[ref55] Rajpurohit Y. S. Sharma D. K. Misra H. S. (2022). Involvement of serine / threonine protein kinases in DNA damage response and cell division in bacteria. Res. Microbiol. 173:103883. doi: 10.1016/j.resmic.2021.103883, PMID: 34624492

[ref56] Roset M. S. Fernández L. G. DelVecchio V. G. Briones G. (2013). Intracellularly induced cyclophilins play an important role in stress adaptation and virulence of *brucella abortus*. Infect. Immun. 81, 521–530. doi: 10.1128/IAI.01125-12, PMID: 23230297 PMC3553818

[ref57] Roy J. Rineau F. De Boeck H. J. Nijs I. Pütz T. Abiven S.. (2021). Ecotrons: powerful and versatile ecosystem analysers for ecology, agronomy and environmental science. Glob. Change Biol. 27, Issue 7, 1387–1407. doi: 10.1111/gcb.15471, PMID: 33274502 PMC7986626

[ref58] Ruiz N. Lavelle P. Jiménez J. (2008). Food and agriculture Organization of the United Nations soil macrofauna field manual technical level

[ref59] Salomon M. J. Cavagnaro T. R. (2022). Healthy soils: the backbone of productive, safe and sustainable urban agriculture. J. Clean. Prod. 341:130808. doi: 10.1016/j.jclepro.2022.130808

[ref60] Santos-Medellín C. Edwards J. Liechty Z. Nguyen B. Sundaresan V. (2017). Drought stress results in a compartment-specific restructuring of the rice root-associated microbiomes. MBio 8, 10–1128. doi: 10.1128/mBio.00764-17, PMID: 28720730 PMC5516253

[ref61] Scheu S. Ruess L. Bonkowski M. (2005). “Interactions between microorganisms and soil micro- and mesofauna” in Microorganisms in soils: roles in genesis and functions. ed. Bonkowski M. (Heidelberg Germany: Springer-Verlag), 253–275.

[ref62] Scholle G. Wolters V. Joergensen R. G. (1992). Effects of mesofauna exclusion on the microbial biomass in two moder profiles. Biol. Fertil. Soils 12, 253–260. doi: 10.1007/BF00336040

[ref63] Seaton F. M. Reinsch S. Goodall T. White N. Jones D. L. Griffiths R. I.. (2022). Long-term drought and warming alter soil bacterial and fungal communities in an upland heathland. Ecosystems 25, 1279–1294. doi: 10.1007/s10021-021-00715-8

[ref64] Sebai T. E. Abdallah M. (2022). Role of microorganisms in alleviating the abiotic stress conditions affecting plant growth. Available online at: www.intechopen.com (Accessed November 18, 2024).

[ref65] Setälä H. Laakso J. Mikola J. Huhta V. (1998). Functional diversity of decomposer organisms in relation to primary production. Appl. Soil Ecol. 9, 25–31. doi: 10.1016/S0929-1393(98)00049-3

[ref66] Sheik C. S. Beasley W. H. Elshahed M. S. Zhou X. Luo Y. Krumholz L. R. (2011). Effect of warming and drought on grassland microbial communities. ISME J. 5, 1692–1700. doi: 10.1038/ismej.2011.32, PMID: 21451582 PMC3176507

[ref67] Shemesh M. Chaia Y. (2013). A combination of glycerol and manganese promotes biofilm formation in *Bacillus subtilis* via histidine kinase KinD signaling. J. Bacteriol. 195, 2747–2754. doi: 10.1128/JB.00028-13 23564171 PMC3697245

[ref68] Solana García Andrea F. F. A. (2021). Andrea Solana García Tutores: Ana María Fita Fernández Óscar Vicente Meana María de la O Plazas Ávila.

[ref69] Thakur M. P. Geisen S. (2019). Trophic regulations of the soil microbiome. Trends in Microbiol. 27, 771–780. doi: 10.1016/j.tim.2019.04.008 31138481

[ref70] Tsiafouli M. A. Kallimanis A. S. Katana E. Stamou G. P. Sgardelis S. P. (2005). Responses of soil microarthropods to experimental short-term manipulations of soil moisture. Appl. Soil Ecol. 29, 17–26. doi: 10.1016/j.apsoil.2004.10.002

[ref71] Valencia E. Gross N. Quero J. L. Carmona C. P. Ochoa V. Gozalo B.. (2018). Cascading effects from plants to soil microorganisms explain how plant species richness and simulated climate change affect soil multifunctionality. Glob. Chang. Biol. 24, 5642–5654. doi: 10.1111/gcb.14440, PMID: 30239067

[ref72] Wagg C. Bender S. F. Widmer F. Van Der Heijden M. G. A. (2014). Soil biodiversity and soil community composition determine ecosystem multifunctionality. Proc. Natl. Acad. Sci. USA 111, 5266–5270. doi: 10.1073/pnas.1320054111, PMID: 24639507 PMC3986181

[ref73] Watzinger A. Prommer J. Spiridon A. Kisielinska W. Hood-Nowotny R. Leitner S.. (2023). Functional redundant soil fauna and microbial groups and processes were fairly resistant to drought in an agroecosystem. Biol. Fertil. Soils 59, 629–641. doi: 10.1007/s00374-023-01728-2

[ref74] Weißbecker C. Schnabel B. Heintz-Buschart A. (2020). Dadasnake, a snakemake implementation of DADA2 to process amplicon sequencing data for microbial ecology. GigaScience 9:giaa135. doi: 10.1093/gigascience/giaa135, PMID: 33252655 PMC7702218

[ref75] Wu T. Su F. Han H. Du Y. Yu C. Wan S. (2014). Responses of soil microarthropods to warming and increased precipitation in a semiarid temperate steppe. Appl. Soil Ecol. 84, 200–207. doi: 10.1016/j.apsoil.2014.07.003

[ref76] Yang N. Nesme J. Røder H. L. Li X. Zuo Z. Petersen M.. (2021). Emergent bacterial community properties induce enhanced drought tolerance in Arabidopsis. Npj Biofilms Microb. 7:82. doi: 10.1038/s41522-021-00253-0, PMID: 34795326 PMC8602335

[ref77] Yang J. Schrader S. Tebbe C. C. (2024). Legacy effects of earthworms on soil microbial abundance, diversity, and community dynamics. Soil Biol. Biochem. 190:109294. doi: 10.1016/j.soilbio.2023.109294

