# Supplementary material for: Functional diversity of soil macrofauna may contribute to microbial community stabilization under drought stress
Source: Front Microbiol. 2025 Jun 13;16:1597272. doi: 10.3389/fmicb.2025.1597272 (PMC12202550; doi:10.3389/fmicb.2025.1597272)

# Functional diversity of soil macrofauna may contribute to microbial community stabilization under drought stress

Diana Morales-Fonseca [1,2], Sandra Barantal [3,4], François Buscot [1], Stephan Hättenschwiler [4], Alexandru Milcu [3,4], Johanne Nahmani [4], Emmanuel S. Gritti [3], Kezia Goldmann [1] and Luis Daniel Prada-Salcedo [1]*

1 Department of Soil Ecology, UFZ-Helmholtz Centre for Environmental Research, Halle (Saale), Germany,

2 Faculty of Engineering, Department Chemical Engineering, Universidad de América, Bogotá, Colombia,

3 Ecotron Européen de Montpellier, CNRS, Campus Baillarguet, Montferrier-sur-Lez, France,

4 CEFE, Univ Montpellier, CNRS, EPHE, IRD, Montpellier, France

**Suplementary material**

Figure S1: Design of the experiment and rarefaction curves for different sampling points from 2018 to 2020.

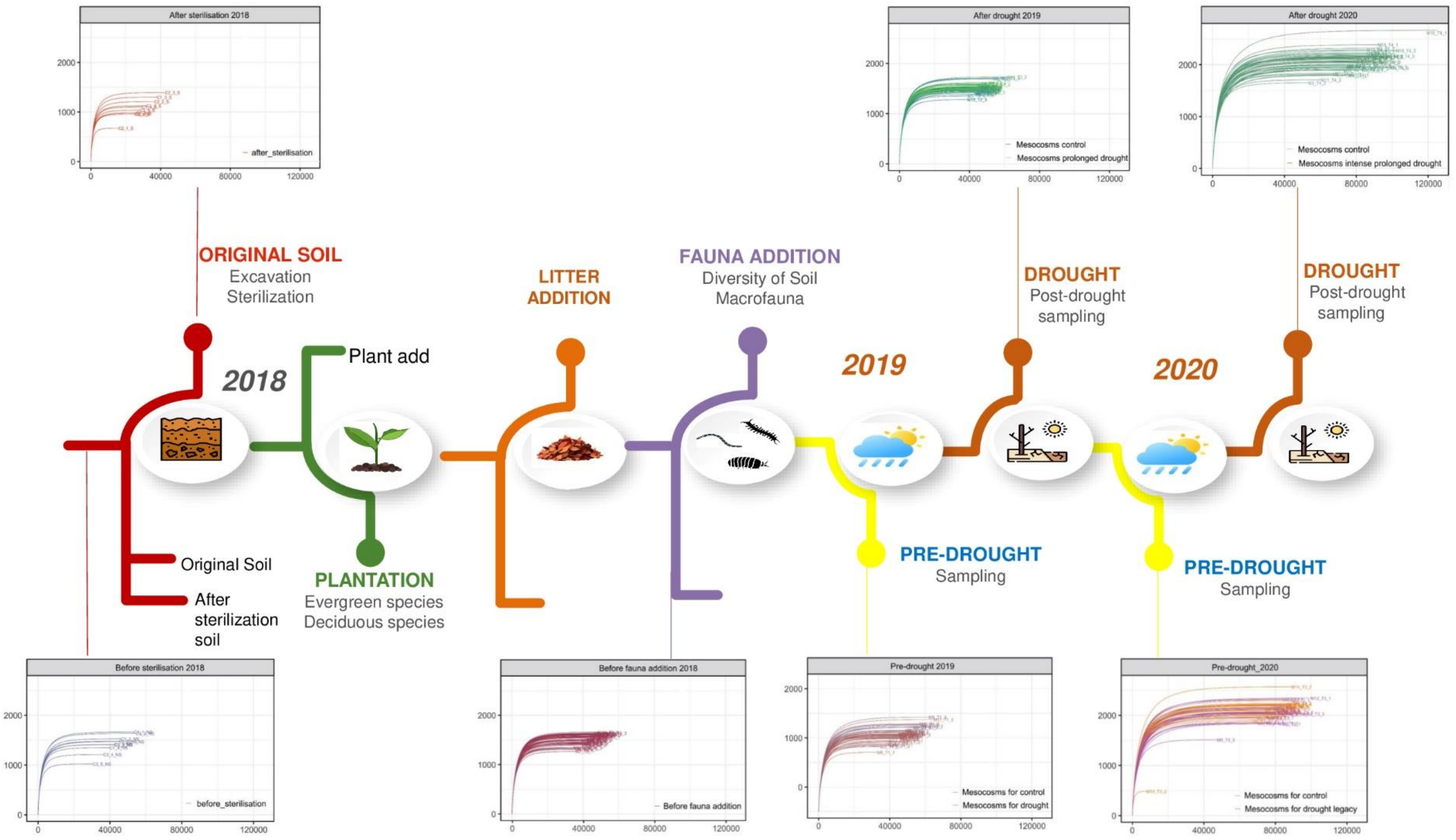

Figure S2: Species indicator for taxa under Control Vs. Drought treatments under two macrofauna functional diversity levels.

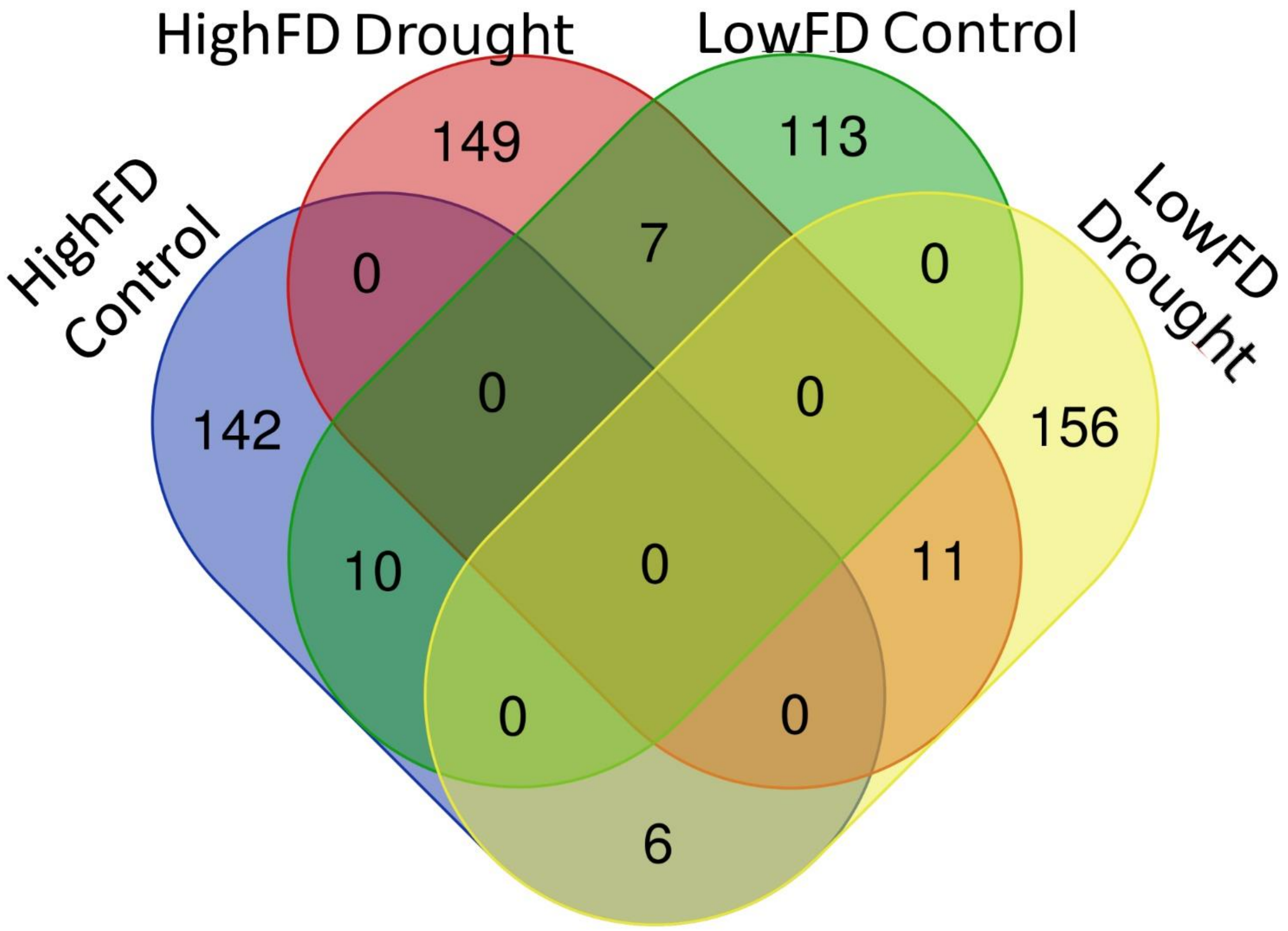

Supplement: Supplementary file 1 [file Data_Sheet_1.pdf]
